# Early User Experiences of CareMobi: mHealth Application for Informal Caregivers and Care Coordination

**DOI:** 10.1093/geroni/igaf122.3750

**Published:** 2025-12-31

**Authors:** Ashley Wei, Laura Peralta, Tina Sadarangani

**Affiliations:** Royal College of Surgeons in Ireland, Edmonton, Alberta, Canada; New York University, New York, New York, United States; New York University, New York, New York, United States

## Abstract

As care of older adults continues to shift toward community and home-based settings, many responsibilities fall to informal caregivers. Despite this, few comprehensive digital tools exist to support their needs. We evaluated early user satisfaction and perceived utility of CareMobi, a mobile application designed to support caregiving for medically complex adults. A cross-sectional post-download survey was distributed to eligible users via the CareMobi newsletter, assessing satisfaction, ease of use, most helpful features, and desired improvements. Users could also provide demographic information, describe how they discovered the app, and share real-world use cases. Informal caregivers most valued features that supported communication and organization. The top 3 features identified were: appointments and calendar tools, health tracking (e.g. vital signs, nutrition, daily updates, etc.), and secure file storage. Overall, 82% of respondents reported being satisfied (37%) or very satisfied (45%) with the app and 76% found CareMobi easy or very easy to use. The most frequently requested feature was a provider contact list (n = 4). Open-ended responses highlighted three key benefits associated with app use: enhanced communication with family members and collaboration with healthcare providers, streamlined information management, and improved health tracking and scheduling. Early users reported that CareMobi reduces caregiver burden by centralizing care management. These initial findings underscore the potential of mHealth solutions to support informal caregivers of older adults and inform future development priorities, as well as the need to integrate these into clinical practice.

